# Species composition, blood meal hosts and *Plasmodium* infection rates of *Anopheles* mosquitoes in Ghibe River Basin, southwestern Ethiopia

**DOI:** 10.1186/s13071-019-3499-3

**Published:** 2019-05-23

**Authors:** Dejene Getachew, Teshome Gebre-Michael, Meshesha Balkew, Habte Tekie

**Affiliations:** 1 Department of Biology, Dire Dawa University, P. O. Box 1362, Dire Dawa, Ethiopia; 2 Department of Zoological Sciences, Addis Ababa University, P. O. Box 1176, Addis Ababa, Ethiopia; 3 Aklilu Lemma Institute of Pathobiology, Addis Ababa University, P. O. Box 1176, Addis Ababa, Ethiopia; 4 Abt Associates, PMI VectorLink Ethiopia Project, Addis Ababa, Ethiopia

**Keywords:** *Anopheles gambiae* (*s.l.*), Bovine blood index, Darge, Ghibe, Human blood index

## Abstract

**Background:**

Vector control interventions using long-lasting insecticidal nets (LLINs) and indoor residual spraying (IRS) are commonly practiced tools for the control of malaria in Ethiopia. In order to evaluate the effectiveness of these control interventions, and understand the prevailing malaria vectors, their incrimination in disease transmission, and their resting and feeding behavior, we set out to identify the *Anopheles* species, their blood meal sources, and entomological inoculation rate (EIR) in Ghibe and Darge within the Ghibe River basin, southwestern Ethiopia.

**Methods:**

Adult *Anopheles* mosquitoes were sampled both indoors and outdoors from January 2015 to October 2016 using Centers for Disease Control and Prevention (CDC) light traps, pyrethrum spray catch (PSC), artificial pit shelters and mouth aspirators. Mosquito species were morphologically identified, and their blood meal sources and malaria sporozoite rates were assessed using enzyme-linked immunosorbent assays.

**Results:**

In total, 13 species of *Anopheles* mosquitoes were identified, among which *Anopheles gambiae* (*s.l.*) was the predominant species: 87.9 and 67.7% in Ghibe and Darge, respectively. The mean density of *An. gambiae* (*s.l.*) collected per night using CDC light traps was 1.8 and 0.7 outdoors and indoors, respectively, in Ghibe, and 0.125 and 0.07 indoors and outdoors, respectively, in Darge. *Anopheles* mosquito abundance was higher in houses near the river than in houses far from the river in both study sites. Among *Anopheles* mosquitoes sampled using CDC light trap catches, 67.6% were unfed and the indoor and outdoor human blood indices of *An. gambiae* (*s.l.*) were 58.4 and 15.8%, respectively in Ghibe, while in Darge, they were 57.1 and 50%, respectively. Sporozoite rates were 0.07% for *P. vivax* and 0.07% for *P. falciparum* in Ghibe and zero in Darge. In Ghibe, the overall EIRs for *P. falciparum* and *P. vivax* were zero and 8.4 infective bites/person/year, respectively, in 2015, while zero and 5.4 infective bites/person/year for *P*. *vivax* and *P. falciparum*, respectively, in 2016. No *Plasmodium-*positive *Anopheles* mosquitoes were identified from Darge.

**Conclusions:**

*Anopheles gambiae* (*s.l.*), the principal vector of malaria in Ethiopia was the most abundant species both indoors and outdoors, fed both on human and cattle blood and occurred at higher frequencies near rivers. *Anopheles gambiae* (*s.l.*) that were circumsporozoite-positive for *Plasmodium* species were collected from Ghibe, but not Darge.

## Background

Malaria has an overwhelming impact on people’s health and livelihoods with an estimated 216 million cases and 445,000 deaths globally in 2016 and about 91% of all malaria deaths being in Africa [1]. In Ethiopia, malaria is a major public health problem with variable transmission and occurrence. Malaria transmission is seasonal and shows variation in its endemicity in the country due to its large diversity in altitude, rainfall, and population movement [2].

In Ethiopia, approximately 68% of the population lives in malaria endemic regions. It has been estimated that 2.8 million cases and 4900 deaths occurred because of malaria in 2015. In Ethiopia, *Plasmodium falciparum* accounts for 64% of the malaria cases while *Plasmodium vivax* accounts for 36% [2, 3], but these percentages might not be constant because of the high degree of seasonal variation in *Plasmodium* species [4–7]. Lowland areas are endemic to malaria while highlands and highland fringe areas are prone to epidemics associated with unusually high minimum temperature together with a lack of immunity in populations [8]. Reports have shown that the number of malaria cases and deaths declined after the scale-up of deployment of artemisinin-based combination therapy (ACT), IRS and wide distribution of LLINs [9, 10].

*Anopheles arabiensis*, a member of *An. gambiae* (*s.l.*), is the principal malaria vector in Ethiopia, whereas *Anopheles pharoensis*, *Anopheles funestus*, and *Anopheles nili* are considered secondary vectors [3, 11]. Understanding the biting and resting habits of *Anopheles* mosquitoes is essential for the implementation of effective vector control interventions [12].

In Ethiopia, malaria vector control relies heavily on IRS and the distribution of LLINs [2]. Indoor residual spraying and LLINs are intradomiciliary-based control measures effective for vectors that closely depend on humans for feeding and resting inside houses [13]. The presence of outdoor feeding and resting *Anopheles* mosquitoes, which cannot be targeted with IRS and LLINs, has resulted in failure of malaria elimination [13]. The presence of these insecticide-based interventions may lead to shifts in the biting behavior of the vectors from indoor to outdoor, from human to animal, and from early before bed time or late at night (dawn) [13–16].

Factors such as the expansion of irrigation schemes [17, 18], construction of large dams [19], asymptomatic subpatent *Plasmodium* carriage in low endemic or pre-elimination transmission settings [20], vector resistance to insecticides [21–23] and drug resistance [24, 25] might pose problems in control and eventual elimination of malaria in Ethiopia. To achieve malaria elimination, both escalation of the current control methods and development of novel interventions to interrupt transmission are needed. Planning and implementation of vector interventions require an understanding of the biology of the important malaria vectors [26].

Thus far, no studies have been conducted on *Anopheles* mosquito species composition, resting behavior, blood meal sources and their disease transmission potential in the study area. An understanding of the local malaria vector species, resting sites, blood meal sources, and their disease incrimination is extremely vital for malaria control and its elimination. Hence, the objective of this study was to identify the *Anopheles* mosquito species, their resting behavior, blood meal sources and the EIR in the Ghibe River basin, southwestern Ethiopia.

## Methods

### Study area

**Fig. 1:**
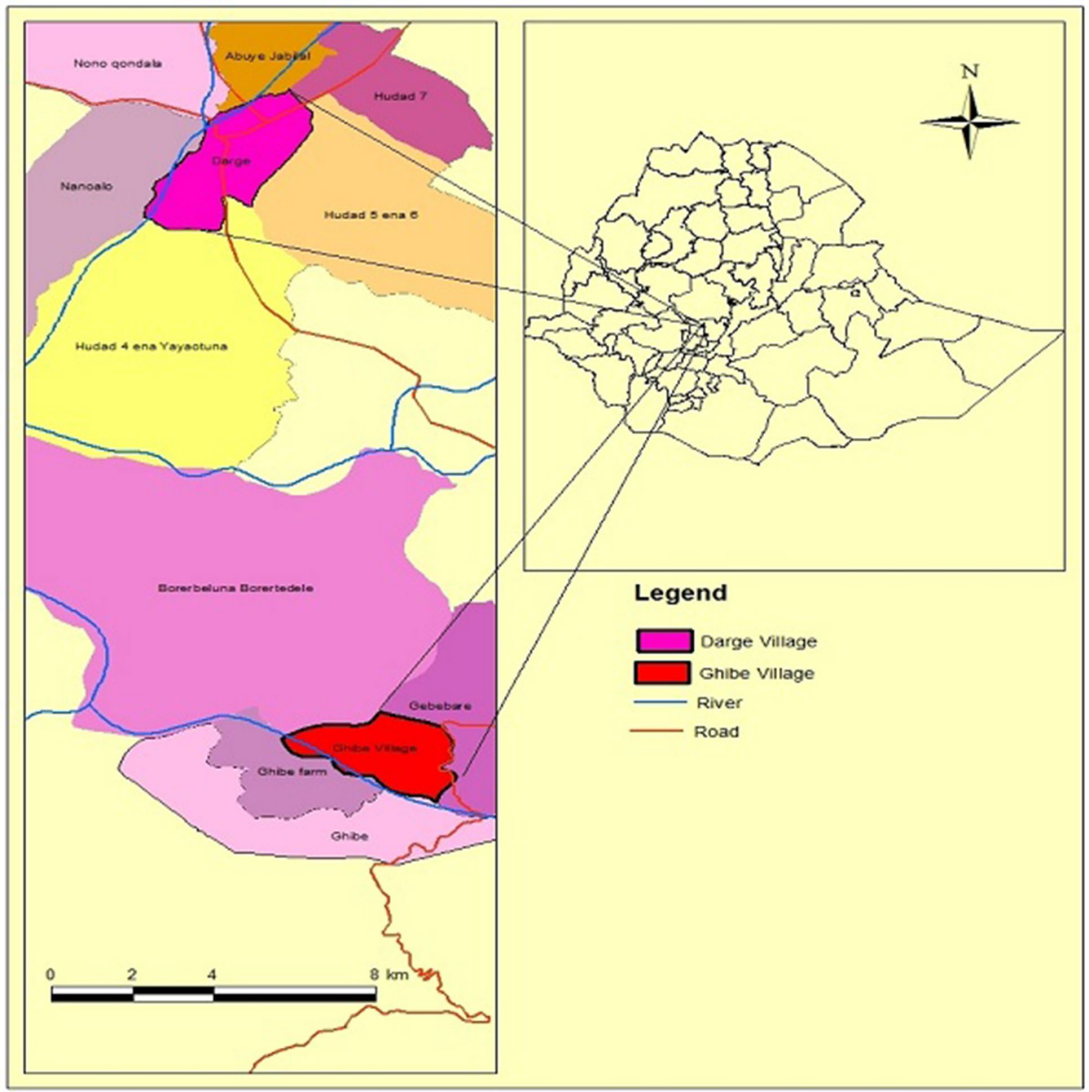
Map of the study area

The study was conducted in Ghibe and Darge study sites located within the Ghibe River Basin, in southwestern Ethiopia in Abeshge district, Guraghe Zone, Southern Nations Nationalities and Peoples Regional State (Fig. 1). The zonal and Abeshge district town (Wolkite) is located 158 km southwest of Addis Ababa. Based on information obtained from the district health office, the area is endemic to malaria. In each study site, there are perennial rivers. Ghibe [8°14′N, 37°33′E, altitude 1080–1134 m above sea level (masl)] is located 30 km south of Wolkite near the Ghibe River. In 2016, Ghibe had 420 households with 2167 total inhabitants of whom 1105 were male and 1062 were female (Abeshge district health office, unpublished report). Darge (8°24′N, 37°31′E, altitude 1500–1800 masl) is located 42 km west of Wolkite and 52 km from Ghibe kebele on the outskirts of Darge town. The Darge River crosses Darge town and serves as one of the tributaries of the Ghibe River. In 2016, Darge had 731 households with 3518 inhabitants, of whom 1724 were male and 1794 were female (Abeshge district health office, unpublished report). In Ghibe, the majority of houses were built with thatched roofs and wooden walls plastered with mud, while in Darge they were constructed with thatched or corrugated iron roofs and wooden walls plastered with mud. The average number of occupants per house in Ghibe and Darge were 5.2 and 4.8, respectively. The human to cattle ratio in Ghibe and Darge were 0.4 and 1.8, respectively (Abeshge District Agricultural Development Office, unpublished data). Ghibe has an annual average rainfall of 625 mm and Darge has 1022 mm (National Meteorological Agency, unpublished report). There was application of IRS on a yearly basis before the onset of the main rainy season, and the residents own LLINs (PermaNet^®^ 2.0) distributed free of charge by the Ministry of Health.

### Adult mosquito collections

Adult *Anopheles* mosquitoes were collected monthly using CDC light traps, PSC, and pit shelter from each study site from January 2015 to February 2016 and April 2016 to October 2016 over 21 months. Houses were first stratified into near to (< 500 m) and far from (> 500 m) Ghibe and Darge rivers at Ghibe and Darge study sites, respectively. Sixteen houses (eight houses near to the river and eight houses far from the river) were selected at random for CDC light traps (BioQuip Products, Rancho Dominguez, CA, USA) for collection of host-seeking mosquitoes indoors and outdoors. In each house, light traps were set at 1–1.5 m above the ground, close to the foot of an occupant sleeping under treated bednets [27]. Collections using CDC light traps were conducted starting from June 2015. For outdoor collection, light traps were hung near houses or in cattle sheds if cattle were available around homesteads. Traps were set for 12 h (18:00 h in the evening to 6:00 h in the morning) for two sequential nights each month for a total of 1184 trap-nights of which 672 were indoors and 512 were outdoors in each study site.

Pyrethrum spray catches and pit shelters were used to collect indoor resting and outdoor resting mosquitoes, respectively [24]. Ten houses (five houses near and five houses far from the river) were selected for spray collection for 21 months in each study site. Before the application of insecticides, all food items, drinking water and domestic animals were moved out from the house. All holes and eaves were closed with pieces of cloth. A white sheet of cloth was spread covering all the floor and furniture within the room where people had slept the previous night. Doors and windows were closed, and Baygon aerosol (prallethrin 0.10% w/w, permethrin 0.10% w/w; Saudi Johnson Co., Racine, WI, USA) was sprayed starting from the outside near the eaves of roof and within the house. After 10 min, the sheet was moved out and knocked down mosquitoes were collected using forceps [28]. All collections were made in the morning (6:30 to 9:00 h). Four pit shelters (two near to the river and two far away from the river) were prepared under shade in the backyards of the households in each study site as described in Silver [29]. Mosquitoes were collected from the small cavities and from the wall of the pit itself using a hand held torch and mouth aspirator from 6:30 to 9:30 h in the morning.

Houses were purposively selected to collect blood-fed mosquitoes, by aspiration using hand-held mouth aspirator and battery-operated torch in the morning from 7:00 to 9:00. In Ghibe, livestock were kept outdoors in separate enclosures also constructed with a thatched roof and wall shelters. In Darge, it was common to keep livestock in houses with humans at night.

### Mosquito identification

Female *Anopheles* mosquitoes were counted and sorted to abdominal conditions as unfed, freshly fed, half gravid, and gravid, and morphologically identified under stereomicroscope using morphological identification keys of Gillies and Coetzee [30]. The abdomens of freshly blood-fed *Anopheles* mosquitoes were used for blood meal analysis.

### Blood meal analysis

Freshly blood-fed *Anopheles* mosquitoes were cut transversally between the thorax and abdomen beneath a dissecting microscope at 10–20× magnification. Blood meal sources were then identified using an enzyme-linked immunosorbent assay (ELISA) following the procedure described by Beier et al. [31].

### Malaria sporozoite ELISA

Heads and thoraces of female *Anopheles* mosquitoes were processed for the detection of circumsporozoite proteins (CSP) of *P. falciparum* and *P. vivax* 210 (Pv-210) and *P. vivax* 247 (Pv-247) sporozoites using an ELISA following the procedure described in Beier et al. [32].

### Data analysis

The monthly abundance of *Anopheles* mosquitoes in different collection methods was expressed with descriptive statistics using percentages. The monthly average density of *Anopheles gambiae* (*s.l.*) using CDC light traps, PSC and pit shelters were expressed as the total number of mosquitoes collected per total monthly catches for each sampling method. Correlation analysis was used to estimate the association between *An. gambiae* (*s.l.*) densities with rainfall. A Chi-square test was used to compare the abundance of *Anopheles* mosquitoes using CDC light traps between near and far houses from the river and between indoor and outdoor catches. Pit shelter and PSC samples were not included in this analysis because *An. gambiae* (*s.l.*) was the only species identified in these collection methods. The human blood index (HBI) and bovine blood index (BBI) were calculated as the ratio of blood-fed mosquitoes that had fed on humans and cattle, respectively, to the total tested. Mixed blood meal source was the ratio of blood-fed mosquitoes that had fed on both human and cattle blood to the total tested represented as a percentage. Unknown blood meal source was the ratio of blood-fed mosquitoes which contained unknown blood sources to the total tested [33] and expressed as a percentage. A Chi-square test was used to compare the differences in the HBI and BBI between indoor and outdoor collected *An. gambiae* (*s.l.*).

The sporozoite rate (SRs) is the number of mosquitoes found positive to CSP antigens divided by the total number of mosquitoes examined, expressed as a percentage [28]. The daily EIR was estimated based on CDC light trap catches and was calculated as 1.605 × (number of sporozoite positive ELISAs/ number of mosquitoes tested) × (number of mosquitoes collected by CDC light trap/ number of CDC light trap catches) [34]. EIR was not estimated for mosquitoes collected with other collection methods because no CSP positive mosquitoes were identified. Monthly EIR was obtained by multiplying the daily EIR with the number of respective days of that month [35]. The monthly EIRs in each study site were summed up to calculate the annual EIR [36]. Data were analyzed using IBM SPSS statistics for Windows v.20.0 (IBM, Armonk, NY, USA). In all tests, values were considered significantly different if *P* < 0.05.

## Results

### Composition of the *Anopheles* mosquitoes

The species composition of *Anopheles* mosquitoes is depicted in Table 1. In total, 13 species of *Anopheles* mosquitoes, including *An. gambiae* (*s.l.*), *Anopheles coustani*, *Anopheles pretoriensis*, *Anopheles demeilloni*, *Anopheles rupicolus*, *Anopheles christyi*, *An. pharoensis*, *An. nili* and *Anopheles rivulorum* were collected in both Ghibe and Darge study sites, whereas *Anopheles tenebrosus*, *Anopheles ardensis*, *Anopheles natalensis*, and *Anopheles zeimanni* were recorded only in Ghibe. *Anopheles gambiae* (*s.l.*) was the predominant species in both study sites (87.9% in Ghibe and 67.7% in Darge) followed by *An. coustani* (5.4% in Ghibe and 14.3% in Darge) (Table 1).

**Table 1:** *Anopheles* species composition in the two study sites (January 2015 to October 2016)

Study site	Species	Indoor					Outdoor					Total (%)
		CDC		PSC		MA inside houses	CDC		Pit shelter		MA cattle shed	
		Near (%)	Far (%)	Near (%)	Far (%)		Near (%)	Far (%)	Near (%)	Far (%)		
Ghibe	*An. gambiae* (*s*.*l.*)	390 (92.9)	47 (74.6)	35 (100)	1 (100)	119 (100)	807 (83.4)	113 (56.8)	131 (99.2)	12 (100)	495 (99.6)	2150 (87.9)
	*An. coustani*	6 (1.4)	12 (19.1)	–	–	–	67 (6.9)	44 (22.1)	–	–	1 (0.2)	130 (5.4)
	*An. pretoriensis*	4 (1.0)	–	–	–	–	27 (2.8)	14 (7.0)	1 (0.8)	–	–	46 (1.9)
	*An. demeilloni*	10 (2.4)	1 (1.6)	–	–	–	12 (1.2)	3 (1.5)	–	–	–	26 (1.1)
	*An. rupicolus*	3 (0.7)	–	–	–	–	16 (1.7)	8 (4.0)	–	–	–	27 (1.1)
	*An. christyi*	1 (0.2)	–	–	–	–	10 (1.0)	8 (4.0)	–	–	–	19 (0.8)
	*An. pharoensis*	5 (1.2)	1 (1.6)	–	–	–	11 (1.1)	1 (0.5)	–	–	–	18 (0.7)
	*An. tenebrosus*	–	–	–	–	–	12 (1.2)	5 (2.5)	–	–	–	17 (0.7)
	*An. ardensis*	–	2 (3.2)	–	–	–	3 (0.3)	1 (0.5)	–	–	–	6 (0.2)
	*An. natalensis*	–	–	–	–	–	–	1 (0.5)	–	–	–	1 (0.1)
	*An. ziemanni*	–	–	–	–	–	1 (0.1)	–	–	–	–	1 (0.1)
	*An. nili*	–	–	–	–	–	2 (0.2)	–	–	–	–	2 (0.1)
	*An. rivulorum*	1 (0.2)	–	–	–	–	–	–	–	–	1 (0.2)	2 (0.1)
	Unidentified	–	–	–	–	–	–	1 (0.5)	–	–	–	1 (0.1)
	Total	420 (100)	63 (100)	35 (100)	1 (100)	119 (100)	968 (100)	199 (100)	132 (100)	12 (100)	497 (100)	2446 (100)
Darge	*An. gambiae* (*s*.*l.*)	60 (84.5)	24 (96.0)	2 (100)	1 (100)	–	20 (26.7)	28 (90.3)	9 (100)	5 (100)	2 (50)	151 (67.7)
	*An. coustani*	3 (4.2)	–	–	–	–	25 (33.3)	2 (6.5)	–	–	2 (50)	32 (14.3)
	*An. pretoriensis*	1 (1.4)	–	–	–	–	2 (2.7)	–	–	–	–	3 (1.3)
	*An. demeilloni*	5 (7.1)	–	–	–	–	8 (10.7)	–	–	–	–	13 (5.8)
	*An. rupicolus*	–	1 (4.0)	–	–	–	12 (16.0)	–	–	–	–	13 (5.8)
	*An. christyi*	1 (1.4)	–	–	–	–	1 (1.3)	–	–	–	–	2 (0.9)
	*An. pharoensis*	–	–	–	–	–	1 (1.3)	–	–	–	–	1 (0.4)
	*An. nili*	–	–	–	–	–	6 (8.0)	–	–	–	–	6 (2.7)
	*An. rivulorum*	1 (1.4)	–	–	–	–	–	1 (3.2)	–	–	–	2 (0.9)
	Total	71 (100)	25 (100)	2 (100)	1 (100)	–	75 (100)	31 (100)	9 (100)	5 (100)	4 (100)	223 (100)

Among the collection methods used, all of the identified *Anopheles* species were represented in CDC light trap collections. Only *An. gambiae* (*s.l.*) mosquitoes were collected by PSC and hand collections with mouth aspirators from inside houses. *Anopheles gambiae* (*s.l.*) and *An. pretoriensis* were collected from pit shelter outdoors, while *An. gambiae* (*s.l.*) and *An. coustani* were collected using mouth aspirators from cattle sheds. All identified species were collected from indoors and outdoors, except *An. tenebrosus*, *An. natalensis*, *An. ziemanni* and *An. nili*, which were sampled only outdoors. About 69% of *Anopheles* mosquitoes were collected using CDC light traps (Table 1).

**Fig. 2:**
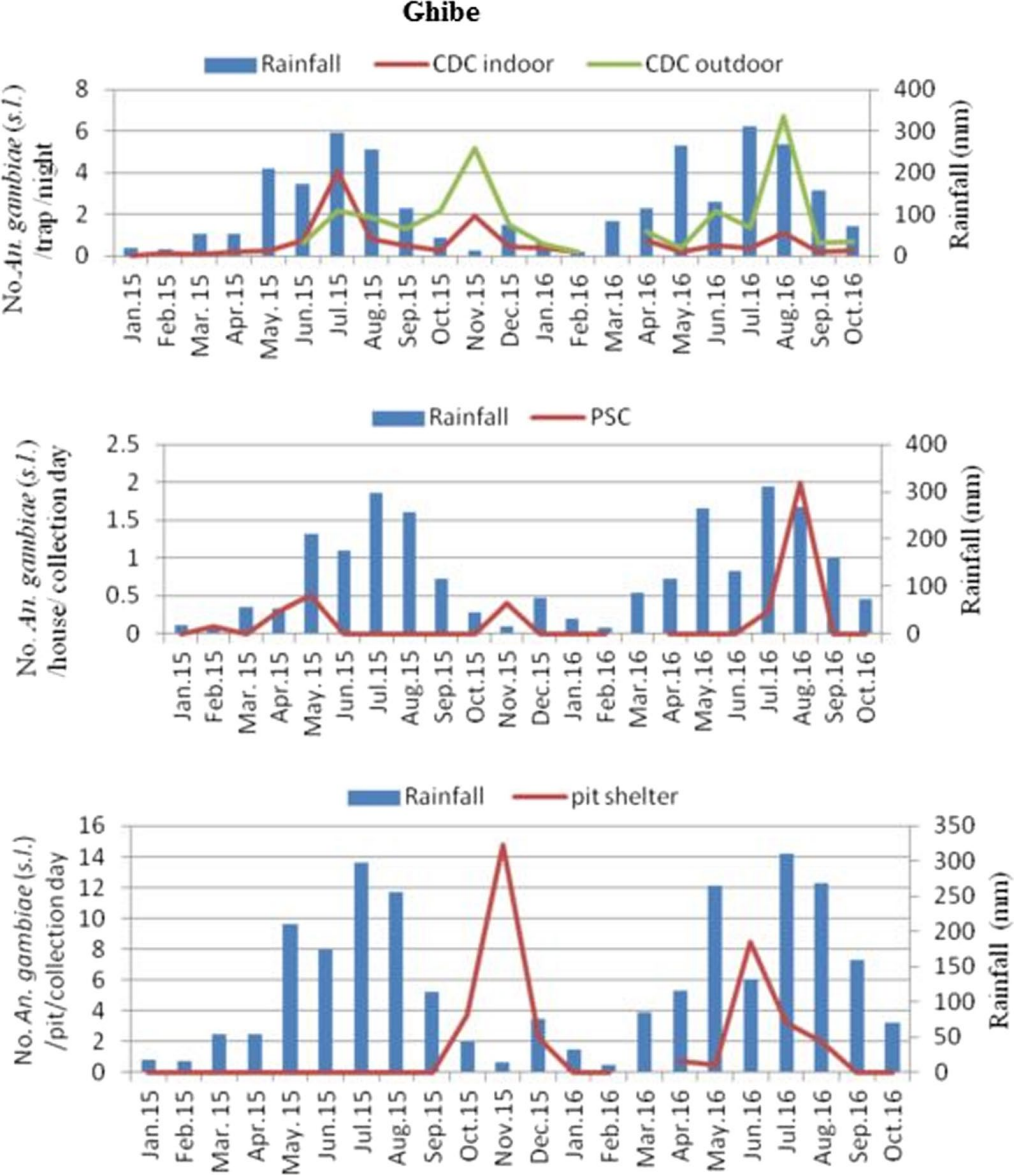
Monthly density of *An. gambiae* (*s.l.*) and its association with rainfall (in mm) in the Ghibe study site

**Fig. 3:**
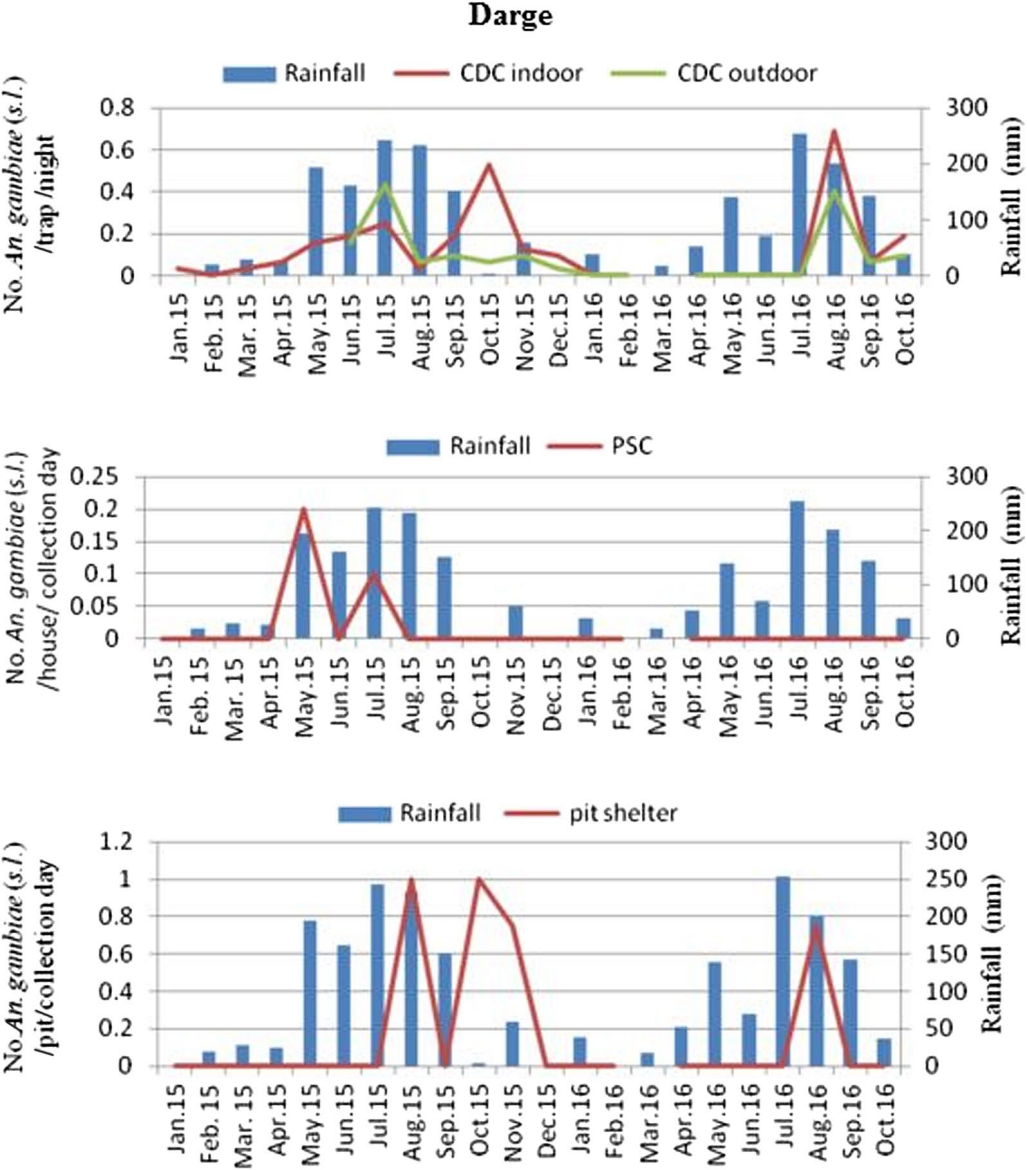
Monthly density of *An. gambiae* (*s.l.*) and its association with rainfall (in mm) in the Darge study site

In Ghibe, the mean density of *An. gambiae* (*s.l.*) using CDC light traps was higher outdoors (1.8/CDC light trap/night) than indoors (0.7/CDC light trap/night) (Fig. 2). The largest proportions of monthly densities of *An*. *gambiae* (*s.l.*) using CDC light traps indoors were 4.1/CDC light trap/night and in outdoor catches were 6.7/CDC light trap/night. The density of this species in PSC was higher in August 2016 (2/house/day) and in pit shelter it was in November 2015 (14.8/pit shelter/day). In Darge, the mean density of *An. gambiae* (*s.l.*) was 0.125/CDC light trap/night indoors and 0.07/CDC light trap/night outdoors. The largest proportions of monthly density of *An*. *gambiae* (*s.l.*) using CDC light trap indoors was 0.69/CDC light trap/night in August, and in outdoor catches was 0.41/CDC light trap/night in July 2015 and August 2016 (Fig. 3). However, using PSC and pit shelters, only 0.01/house/day and 0.67/pit shelter/day were captured. *Anopheles gambiae* (*s.l.*) density was not significantly correlated with rainfall in Ghibe [CDC light trap *r*_(21)_ = 0.388, *P* = 0.082; PSC *r*_(21)_ = 0.324; *P* = 0.152; pit shelter *r*_(21)_ = − 0.156, *P* = 0.499] and in Darge [CDC light trap *r*_(21)_ = 0.204, *P* = 0.374; PSC *r*_(21)_ = 0.395, *P* = 0.076; pit shelter *r*_(21)_ = − 0.037, *P* = 0.873]. Figures 2, 3 show that mosquito population starts to build up immediately after the rains. Analysis to compare *Anopheles* mosquito densities from near and far houses from the river was based on data collected using CDC light traps from June 2015 to October 2016 due to the start of outdoor collection in June 2015 (Table 2). *Anopheles ardensis*, *An*. *natalensis*, *An*. *ziemanni*, *An*. *nili*, *An*. *rivulorum* and unidentified species were excluded from this analysis because of their low numbers. Among the remaining *Anopheles* mosquitoes sampled in Ghibe, significantly more were caught outdoors (*n* = 1157; 72.0%) than indoors (*n* = 450; 28.0%) (Table 2) (*χ*^2^ = 49.037, *df* = 7, *P* < 0.001) and significantly more *Anopheles* were caught in houses closer to the river (*n* = 1350; 84.0%) than in houses located far from the river (*n* = 257; 16.0%) (*χ*^2^ = 113.038, *df* = 7, *P* < 0.001). Similarly, in Darge, significantly more *Anopheles* mosquitoes were sampled outdoors (*n* = 101; 54.6%) than indoors (*n* = 84; 45.4%) (Table 2) (*χ*^2^ = 39.902, *df* = 4, *P* < 0.001), and significantly more *Anopheles* mosquitoes were caught in houses near to the river (*n* = 127; 68.6%) than far from the river (*n* = 58; 31.4%) (*χ*^2^ = 30.759, *df* = 4, *P* < 0.001).

**Table 2:** Comparison of *Anopheles* abundance collected indoor or outdoor and in houses near or far from the rivers

Study site	Species	CDC light trap catch indoor			CDC light trap catch outdoor		
		Near (%)	Far (%)	Total	Near (%)	Far (%)	Total
Ghibe	*An. gambiae* (*s.l*.)	367 (89.1)	45 (10.9)	412	805 (87.5)	115 (12.5)	920
	*An. coustani*	7 (36.8)	12 (63.2)	19	66 (60.0)	44 (40.0)	110
	*An. demeilloni*	10 (90.9)	1 (9.1)	11	12 (80.0)	3 (20.0)	15
	*An. rupicolus*	3 (100)	–	3	16 (66.7)	8 (33.3)	24
	*An. christyi*	1 (100)	–	1	10 (55.6)	8 (44.4)	18
	*An. pretoriensis*	1 (100)	–	1	27 (65.9)	14 (34.1)	41
	*An. pharoensis*	2 (66.7)	1 (33.3)	3	11 (91.7)	1 (8.3)	12
	*An. tenebrosus*	–	–	–	12 (70.6)	5 (29.4)	17
	Total	391 (86.9)	59 (13.1)	450	959 (82.9)	198 (17.1)	1157
Darge	*An. gambiae* (*s.l*.)	48 (64.0)	27 (36.0)	75	20 (41.7)	28 (58.3)	48
	*An. coustani*	3 (100)	–	3	25 (92.6)	2 (7.4)	27
	*An. demeilloni*	5 (100)	–	5	8 (100)	–	8
	*An. rupicolus*	–	1 (100)	1	12 (100)	–	12
	*An. nili*	–	–	–	6 (100)	–	6
	Total	56 (66.7)	28 (33.3)	84	71 (70.3)	30 (29.7)	101

### Blood-feeding status of adult *Anopheles* mosquitoes

With the exception of 87 (3.3%) *Anopheles* mosquitoes, the abdominal conditions of all were classified into unfed, freshly fed, half gravid and gravid (Table 3). From mosquitoes collected using CDC light trap, among their abdominal conditions identified 67.7% (*n* = 1195) were unfed and only 22.5% (*n* = 398) were blood-fed. From PSC, there were no unfed and 51.3% (*n* = 20) were blood-fed, 30.8% (*n* = 12) were half gravid and 17.9% (*n* = 7) were gravid, but 57.3% (*n* = 355) of mosquitoes collected with mouth aspirator were blood-fed and only 3.9% (*n* = 24) were unfed. Catches from pit shelter showed 38.0% (*n* = 60) were blood-fed but lower proportion (12.7%, *n* = 20) of unfed mosquitoes were collected.

**Table 3:** Abdominal status of *Anopheles* mosquitoes collected by different methods in the study sites

Species	CDC					PSC			Pit shelter				Mouth aspirator				Total
	UF	FF	HG	GR	UN	FF	HG	GR	UF	FF	HG	GR	UF	FF	HG	GR	
*An. gambiae* (*s.l.*)	1060	218	71	79	61	20	12	7	19	60	51	27	24	351	138	103	2301
*An. coustani*	79	72	–	1	10	–	–	–	–	–	–	–	–	3	–	–	162
*An. pretoriensis*	8	35	1	2	2	–	–	–	1	–	–	–	–	–	–	–	49
*An. rupicolus*	15	22	2	–	1	–	–	–	–	–	–	–	–	–	–	–	40
*An. demeilloni*	14	18	2	2	3	–	–	–	–	–	–	–	–	–	–	–	39
*An. christyi*	3	14	–	1	3	–	–	–	–	–	–	–	–	–	–	–	21
*An. pharoensis*	3	7	4	5	–	–	–	–	–	–	–	–	–	–	–	–	19
*An. tenebrosus*	6	9	–	1	1	–	–	–	–	–	–	–	–	–	–	–	17
*An. nili*	2	3	–	–	3	–	–	–	–	–	–	–	–	–	–	–	8
*An. ardensis*	2	1	–	1	2	–	–	–	–	–	–	–	–	–	–	–	6
*An. rivulorum*	2	1	–	–	–	–	–	–	–	–	–	–	–	1	–	–	4
*An. ziemanni*	–	–	–	–	1	–	–	–	–	–	–	–	–	–	–	–	1
*An. natalensis*	–	1	–	–	–	–	–	–	–	–	–	–	–	–	–	–	1
Unidentified	1	–	–	–	–	–	–	–	–	–	–	–	–	–	–	–	1
Total	1195	398	80	92	87	20	12	7	20	60	51	27	24	355	138	103	2669

*Abbreviations*: PSC, Pyrethrum spray sheet collection; MA, mouth aspirator; UF, unfed; FF, fresh fed; HF, half gravid; GR, gravid; UN, abdominal status unknown

### Blood meal sources and blood indices

Human blood was identified from *An. gambiae* (*s.l.*), *An. coustani*, *An. demeilloni* and *An. nili* and no other species of *Anopheles* mosquitoes were identified as having fed on humans (Table 4). Human and cattle mixed blood was detected from *An. gambiae* (*s.l.*) and *An. demeilloni*. More than 35% of the blood meal sources of *An. gambiae* (*s.l.*) collected outdoors using CDC light traps were not known (not fed on humans or cattle) (Table 4).

**Table 4:** Blood meal origins of *Anopheles* mosquitoes collected using CDC light trap and PSC

Study site and species	CDC indoor					CDC outdoor					PSC				
	*n*	HBI (%)	BBI (%)	Mixed (%)	Un (%)	*n*	HBI (%)	BBI (%)	Mixed (%)	Un (%)	*n*	HBI (%)	BBI (%)	Mixed (%)	Un (%)
Ghibe															
*An. gambiae* (*s.l.*)	113	58.0	14.0	4.4	23.0	76	16.0	47.0	1.3	35.5	20	75.0	10.0	5.0	10.0
*An. pretoriensis*	3	–	67.0	–	33.0	29	–	97.0	–	3.5	–	–	–	–	–
*An. coustani*	3	33.0	67.0	–	–	52	–	98.0	–	1.9	–	–	–	–	–
*An. demeilloni*	4	–	100	–	–	9	–	78.0	11.1	11.1	–	–	–	–	–
*An. christyi*	–	–	–	–	–	13	–	100	–	–	–	–	–	–	–
*An. rupicolus*	1	–	100	–	–	12	–	100	–	–	–	–	–	–	–
*An. pharoensis*	1	–	100	–	–	6	–	83.0	–	16.7	–	–	–	–	–
*An. tenebrosus*	–	–	–	–	–	9	–	100	–	–	–	–	–	–	–
*An. ardensis*	–	–	–	–	–	1	–	100	–	–	–	–	–	–	–
*An. natalensis*	–	–	–	–	–	1	–	100	–	–	–	–	–	–	–
Darge															
*An. gambiae* (*s.l.*)	21	14.0	57.0	–	28.6	8	13.0	50.0	–	37.5	–	–	–	–	–
*An. pretoriensis*	1	–	100	–	–	2	–	100	–	–	–	–	–	–	–
*An. coustani*	2	50.0	–	–	50.0	12	–	92.0	–	8.3	–	–	–	–	–
*An. demeilloni*	3	–	100	–	–	1	–	100	–	–	–	–	–	–	–
*An. christyi*	–	–	–	–	–	1	–	100	–	–	–	–	–	–	–
*An. rupicolus*	1	–	100	–	–	8	–	100	–	–	–	–	–	–	–
*An. rivulorum*	1	–	100	–	–	–	–	–	–	–	–	–	–	–	–
*An. nili*	–	–	–	–	–	3	33.0	67.0	–	–	–	–	–	–	–

*Abbreviations*: PSC, pyrethrum spray sheet collection; n, number tested; HBI, human blood index; BBI, bovine blood index; Mixed, human and cattle mixed blood index; Un, unidentified blood meal

In Ghibe, the HBI for *An. gambiae* (*s.l.*) was 58.0% from CDC light trap collections indoor and 16.0% outdoor. The BBI from CDC light trap collections indoor was 14.0% and in outdoor was 47.0%. There was significant difference between indoor and outdoor catches in their blood meal sources (*χ*^2^ = 42.134, *df* = 3, *P* < 0.001). The HBI was 75.0% from PSC. Mixed blood (human and cattle) sources for *An. gambiae* (*s.l.*) using CDC light trap indoor, CDC light trap outdoor and PSC were 4.4, 1.3 and 5.0%, respectively.

In Darge, the HBI of *An. gambiae* (*s.l.*) from CDC light trap collections indoors and outdoors was 14.0 and 13.0%, respectively, while the BBI was higher both indoor (57.0%) and outdoor (50.0%) than that of HBI. However, there was no significant difference between HBI or BBI of *An. gambiae* (*s.l.*) caught indoors and outdoors (*χ*^2^ = 0.216, *df* = 2, *P* = 0.898).

In Ghibe, the BBI of *An. gambiae* (*s.l.*) collected from pit shelters and mouth aspirated from cattle sheds was 83.0 and 68.0%, respectively, and the HBI using mouth aspirators from human dwellings was 74.0% (Table 5). Although the proportion was low, human blood-fed and mixed blood-fed mosquitoes were also identified from pit shelters and cattle sheds. In Darge, all of the tested mosquitoes collected from cattle sheds had fed on cattle.

**Table 5:** Blood meal origins of *An. gambiae* (*s.l.*) mosquitoes collected using pit shelter and mouth aspirator

Collection method	*n*	HBI (%)	BBI (%)	Mixed (%)	Un (%)
Pit shelter	60	3.0	83.0	1.7	11.7
MA human house	58	74.0	14.0	–	12.1
MA cattle shed	291	2.0	68.0	0.7	29.6

*Abbreviations*: MA, mouth aspirator; n, number tested; HBI, human blood index; BBI, bovine blood index; Mixed, human and cattle mixed blood index; Un, unidentified blood meal

In total, 1801 *Anopheles* mosquitoes (1668 from Ghibe and 133 from Darge) belonging to 12 species were tested for *Plasmodium* CSP using ELISA. Of these 1620 (90%) were *An. gambiae* (*s.l.*) (Table 6). Out of the total tested, sporozoites were detected only in two of the *An. gambiae* (*s.l.*) (one *P. vivax*, i.e. Pv-210, and one *P. falciparum*) collected using CDC light traps. All the other *Anopheles* mosquitoes species tested were negative for *Plasmodium* infection. Sporozoite rates were 0.07% for *P. vivax* and 0.07% for *P. falciparum* in *An. gambiae* (*s.l.*) tested in Ghibe and zero in Darge. *Anopheles gambiae* (*s.l.*) tested had overall *P. vivax* and *P. falciparum* sporozoite rates of 0.06% each.

**Table 6:** Sporozoite infection rates of *Anopheles* mosquitoes in two study sites in Ghibe River basin, southwestern Ethiopia (January 2015 to October 2016)

	*An. gambiae* (*s.l.*)	*An. coustani*	*An. pharoensis*	*An. demeilloni*	*An. pretoriensis*	*An. christyi*	*An. rupicolus*	*An. nili*	*An. tenebrosus*	*An. rivulorum*	*An. ardensis*	*An. zeimanni*
Ghibe												
No. tested	1521	74	9	13	14	6	14	2	8	1	5	1
No. PvS+ (%)	1 (0.07)	0	0	0	0	0	0	0	0	0	0	0
No. PfS+ (%)	1 (0.07)	0	0	0	0	0	0	0	0	0	0	0
Darge												
No. tested	99	17	0	8	0	1	4	3	0	1	0	0
No. PvS+ (%)	0	0	0	0	0	0	0	0	0	0	0	0
No. PfS+ (%)	0	0	0	0	0	0	0	0	0	0	0	0
Total												
No. tested	1620	91	9	21	14	7	18	5	8	2	5	1
No. PvS+ (%)	1 (0.06)	0	0	0	0	0	0	0	0	0	0	0
No. PfS+ (%)	1 (0.06)	0	0	0	0	0	0	0	0	0	0	0

*Abbreviations*: PvS+, number *P. vivax* sporozoite positive; PfS+, number *P. falciparum* sporozoite positive; values in parentheses indicate sporozoite rates

In Ghibe, the overall EIRs for *P. falciparum* and *P. vivax* were zero and 8.4 infective bites/person/year, respectively, for the year 2015, while zero and 5.4 infective bites/person/year for *P*. *vivax* and *P. falciparum*, respectively, for the year 2016 (Table 7). No *Plasmodium-*positive *Anopheles* mosquitoes were identified from Darge so that EIR was not analyzed.

**Table 7:** Sporozoite rate and EIR of *An. gambiae* (*s.l*.) in Ghibe study site of Ghibe River basin, southwestern Ethiopia (January 2015 to October 2016)

Month-year	Daily PvSR	Monthly PvEIR	Daily PfSR	Monthly PfEIR
Jan-15	0	0	0	0
Feb-15	0	0	0	0
Mar-15	0	0	0	0
Apr-15	0	0	0	0
May-15	0	0	0	0
Jun-15	0	0	0	0
Jul-15	0	0	0	0
Aug-15	0	0	0	0
Sep-15	0	0	0	0
Oct-15	0.09	8.4	0	0
Nov-15	0	0	0	0
Dec-15	0	0	0	0
2015 total	0.09	8.4	0	0
Jan-16	0	0	0	0
Feb-16	0	0	0	0
Apr-16	0	0	0	0
May-16	0	0	0.1	5.4
Jun-16	0	0	0	0
Jul-16	0	0	0	0
Aug-16	0	0	0	0
Sep-16	0	0	0	0
Oct-16	0	0	0	0
2016 total	0	0	0.1	5.4

*Abbreviations*: PvSR, *P. vivax* sporozoite rate; PvEIR, *P. vivax* entomologic inoculation rate; PfSR, *P*. *falciparum* sporozoite rate; PfEIR, *P. falciparum* entomological inoculation rate, results in bold showed annual EIR

## Discussion

*Anopheles gambiae* (*s.l.*) was the predominant species in the study area followed by *An. coustani*. A study by Tekie [37] in the area also showed that *An. gambiae* (*s.l.*) was the predominant species. Similarly, other studies conducted in Ethiopia showed that *An. arabiensis* [member of *An*. *gambiae* (*s.l.*)] was the most abundant species in the country [23, 38, 39]. However, a study in Edo Kontola village, south-central Ethiopia, found that *An. ziemanni* was the predominant species followed by *An. arabiensis*; these observed differences could be explained by variation in available larval breeding sites that support the development of the former species [40].

More *Anopheles* mosquitoes were sampled outdoors using CDC light traps, pit shelters and mouth aspirators than indoors. The human landing catch in Ghibe Horticulture Development Farm showed *An. gambiae* (*s.l.*) bites human both indoors and outdoors [37]. This outdoor abundance of *Anopheles* mosquitoes could be attributed to females having left houses immediately after feeding indoors [41], increased outdoor biting due to the availability of cattle outside houses [42, 43], vector control interventions targeting indoor biting and resting mosquitoes that induced exophagic and late biting behavior [40, 44–47], or rapid exit after entering houses [13, 47]. The availability of cattle outdoors might enhance outdoor catches using CDC light traps, thus extending the host-seeking flight period, which in turn increases the chance of getting caught in the trap [48]. However, a study conducted in Lake Victoria, Kenya, sampled higher numbers of *Anopheles* mosquitoes indoors than outdoors in villages with low LLIN coverage than those with high coverage [49].

Low densities of *Anopheles* mosquitoes were collected from Darge as compared to Ghibe. The possible reasons could be attributed to the difference in the type of house construction materials [50, 51], variation in proportions of cattle owned by the residents [43], altitudinal variation between the two study sites [38] or availability of breeding habitat during the study period [52]. *Anopheles gambiae* (*s.l.*) density was not significantly correlated with rainfall. This might be attributed to the spraying of houses with insecticides before the onset of the main rain or that the larval habitat could be affected due to heavy rain or formed after rains. Our study also showed that *Anopheles* mosquitoes were more abundant in houses closer to the river (within 500 m distance) than in houses far away from the river. In agreement with our results, a study in western Kenya highlands [50] and Iguhu Village, western Kenya [53], showed that houses located closer to the vector habitats had a significantly higher distribution of adult mosquitoes than those farther away. Residents near to the major vector-breeding sites were also observed to be more affected by malaria in urban Uganda [54] and Adama town, in Ethiopia [55], as compared to residents far from breeding sites.

Our study showed that in Ghibe, HBI was higher from mosquitoes collected indoors from human dwellings, while BBI was higher from outdoor collections, which is in agreement with findings of Hadis et al. [56] and Massebo et al. [57]. In contrast to our result, other studies found that the HBI of *An. arabiensis* collected indoors and outdoors were not different, but BBI was higher among indoor than outdoor collected mosquitoes [33], and mosquitoes collected outdoors had a higher HBI than those collected indoors [58]. It was suggested that people might be bitten more frequently outdoors, or that indoor-biting mosquitoes do not remain inside and instead exit houses after feeding [41, 58] or might also bite in the early evening indoors and outdoors at times when the local people were not protected by LLINs [16, 40, 47]. However, in Darge there was no significant difference between BBI in CDC light trap set indoors and outdoors. It was observed that people share houses with cattle in Darge so that mosquitoes may bite cattle indoors. The presence of domesticated animals in the same houses with humans at night likely plays a key role in blood-feeding from humans and animals by local malaria vectors [59].

The availability of alternative hosts like cattle can significantly affect the resting and feeding preference of *Anopheles* mosquitoes at the household level [43, 49, 57, 59]. A study in costal Kenya showed that the primary malaria vectors have shifted from feeding on humans to animals, coinciding with the mass distribution of LLINs in the area [46, 59]. Animals are considered as a dead end for human malaria pathogens [46, 59] and zooprophylactic agents for zoophagic vectors like *An. arabiensis* [60]. Hence, in areas where *An. arabiensis* (which has zoophagic behavior) is the major malaria vector, the use of cattle treated with insecticides could be helpful for their control [61, 62]. New control tools that target mosquitoes biting outdoors and early at night before people go to bed are urgently required to control malaria in combination with the existing control interventions [14].

The sporozoite rate for *An. gambiae* (*s.l.*) was very low (0.07% for *P. vivax* and *P. falciparum* in Ghibe and zero in Darge) as compared to 1.5 and 0.3% for *P. falciparum* and *P. vivax*, respectively, in the suburbs of Jimma town [63], 1.18% for *P. falciparum* in the Zway area, central Ethiopia [17], and 4.1% in lowland areas around dams in Ethiopia [19]. These observed differences in sporozoite rates could be explained by the variation in sampling season of *Anopheles* mosquitoes in which mosquito collection was during peak malaria transmission season or due to availability of small-scale irrigation or dam construction in the previous studies. In-line with the study conducted in south-central Ethiopia [38], our study revealed that *Plasmodium* infections were detected in *An. gambiae* (*s.l.*) collected at the end of short rainy season (May) and long rainy season (October). However, *An. arabiensis* and *An. pharoensis* were found infected with *P. falciparum* sporozoites both in the dry and short rainy seasons in areas with irrigation scheme of Zway [17].

Our study revealed that the annual EIR was very low (8.4 infective bites/person/year for *P. vivax* and 5.4 infective bites/person/year for *P. falciparum*) in Ghibe and was zero in Darge during the study period. One possible explanation is an extended period of low rainfall during the study period [36]. A recent study showed that EIR of *An. arabiensis* collected near a dam constructed at lowland area was very high, with a value of 129.8 infective bites/person/year [19]. Studies also showed that variation in EIR could be observed between the periphery and the center within the same town [34, 63].

Although malaria prevalence was low in the study area, the annual EIR was not zero infective bites/person/year in Ghibe but zero in Darge. A study in south-central Ethiopia showed that all tested *Anopheles* mosquitoes were negative for *P*. *falciparum* and *P*. *vivax* CSP [15, 39]. In some locations, a few people might be bitten multiple times by mosquitoes and may remain infected, although overall prevalence falls in a population. Most mosquitoes become infected when they bite this group of people and likely not if they bite others [64].

This line of study may further be improved upon in future studies with the incorporation of a window exit trap to study the indoor feeding and resting behavior of *Anopheles* mosquitoes as they leave the houses after feeding and/or resting. For the determination of blood meal sources of *Anopheles* mosquitoes in the study area, they were tested only for identifying human or cattle blood sources, which likely missed other animals that serve as alternative blood meal sources [49].

## Conclusions

*Anopheles gambiae* (*s.l.*), which is considered as the main malaria vector in Ethiopia, was the predominant species in the study area. This species of mosquitoes was collected both indoors and outdoors. Blood meal analysis showed that they fed both on humans and cattle. *Anopheles* mosquitoes were abundant in houses located closer to the river as compared to houses far away from the river. In determining malaria risks, vector control strategies targeted to houses closer to the river could have great importance in malaria reduction. It might be difficult to control malaria with the current vector control interventions due to the availability of malaria vectors both indoors and outdoors. Thus, additional measures need to be considered to effectively reduce their numbers outdoors.

## Data Availability

The data sets generated and/or analyzed during the present study are available from the corresponding author upon reasonable request.

## Abbreviations

CDC: Center for Disease Control and Prevention
BBI: bovine biting index
ELISA: enzyme-linked immunosorbent assay
HBI: human biting index
IRS: indoor residual spraying
LLINs: long-lasting insecticidal nets
WHO: World Health Organization
